# Association between Disease Severity, Heart Rate Variability (HRV) and Serum Cortisol Concentrations in Horses with Acute Abdominal Pain

**DOI:** 10.3390/ani10091563

**Published:** 2020-09-02

**Authors:** Heidrun Gehlen, Maria-Dorothee Faust, Remigiusz M. Grzeskowiak, Dagmar S. Trachsel

**Affiliations:** 1 Equine Clinic, Veterinary Department, Freie University Berlin, 14163 Berlin, Germany; Heidrun.Gehlen@fu-berlin.de (H.G.); doro.faust@gmx.de (M.-D.F.); 2 Large Animal Clinical Science, College of Veterinary Medicine, University of Tennessee, 2407 River Dr, Knoxville, TN 37996, USA; remik.grzeskowiak@gmail.com

**Keywords:** frequency domain, time domain, gastrointestinal diseases, equine, stress

## Abstract

**Simple Summary:**

Acute abdominal pain is a major cause for emergency treatment in horses and associated with a high stress level leading to an increased serum cortisol concentration. Stress can also be assessed by analyzing the heart rate variability (HRV). We investigated whether the stress level was different between horses with different causes of abdominal pain and, therefore, demanding a different treatment strategy. Heart rate, its variability in the time domain analyses, and cortisol level indicated a decrease in the stress level the day after admission and the day of discharge from the hospital in comparison to admission for both conservatively and surgically treated patients. However, such changes, over time, were not seen in horses that were euthanized during the hospitalization. Furthermore, the difference in the parameters measured between horses that were eventually euthanized and those that survived was best visible the day after admission. Therefore, we concluded that HRV can give further important information on the stress level in horses with colic and might be helpful in assessing possible outcome. However, further studies are required to assess the validity of HRV analyses in horses with colic.

**Abstract:**

Heart rate variability (HRV) is a noninvasive technique to detect changes in the autonomous nervous system. It has rarely been investigated in horses with colic. Therefore, the objective was to assess the evolution of HRV parameters and cortisol concentrations in horses with colic. The 43 horses included in this study were categorized into three groups according to the treatment (1, surgical; 2, conservative; 3, euthanized). The HRV and laboratory variables were measured at admission (T1), the day after admission (T2), and at discharge (T3) and compared between groups and over time with an ANOVA with Bonferroni correction. Relationships between the HRV parameters themselves and the laboratory variables was assessed by Pearson correlation coefficients. Evolution of the heart rate (HR) over time, mean normal to normal R intervals (meanNN) and cortisol concentrations indicate a decreased sympathetic stimulation over time in group 1 and 2, in contrast to group 3. For group 3, the meanNN and HR differed significantly to group 2 at T1 and to group 1 and 2 at T2. Treatment induced a change in the HRV and cortisol response in horses managed conservatively or surgically but not in horses that required euthanasia. However, further studies are required to assess the validity of HRV analyses in horses with colic.

## 1. Introduction

Acute abdominal pain (colic) caused by gastrointestinal tract diseases in horses represents a frequent emergency in equine medicine and, depending on the cause of the colic, can be associated with a guarded to poor prognosis [1,2]. The initial exam aims at triaging for therapeutic intervention. However, time for the diagnosis and treatment is limited, especially when a surgical intervention is indicated, due to the dynamic progression of the condition. Surgeries are often associated with higher treatment expenses. Furthermore, the convalescence may take longer [3], and outcomes, survival rate, and future use are affected [4,5]. Therefore, diagnostic tests or procedures helping in decision-making as soon as possible after admission is needed. Available research in horses with acute gastrointestinal diseases has focused on investigating laboratory diagnostic variables (e.g., tumor necrosis factor, serum amyloid A, and plasma lactate). Regarding these variables, the goals were to assess the status of the circulatory volume and the damage to the intestinal wall [6,7,8,9,10] to predict the need for surgery or survival. The heart rate variability (HRV) has only rarely been used so far for this purpose [11].

Analysis of the HRV is a non-invasive technique to assess the activity of the autonomous nervous system [12]. In human medicine, the HRV has shown diagnostic values in assessing the progression of various diseases, for example survival after myocardial infarction [13] or neuropathy in diabetic patients [14]. The HRV has been used in behavioral research in horses for investigating the reaction of the autonomous nervous system to different stressful situations [15,16,17]. Furthermore, it has been utilized to identify dysautonomia in equine grass sickness [18], to assess different pain conditions [19], and to monitor the influence of special surgical investigations on the autonomous nervous system [20]. The analyses of the HRV can be done with two different approaches: the time domain analysis and the frequency domain analysis. The time domain analysis is based on the mathematical analysis of successive RR-intervals. Increasing variation in the successive RR intervals are related to high parasympathetic activity. The frequency domain analysis is based on a power spectral analysis using a fast Fourier transformation and the obtained power specters are decomposed in a high frequency (HF) range and a low frequency (LF) range. The HF represents the parasympathetic activity, whereas the LF and the LF/HF represents the sympathetic-vagal balance [21,22]. Even though the HRV analysis is a noninvasive method to assess stress and painful conditions, studies in horses with signs of acute abdominal pain are sparse [11]. The objective of the present study was to investigate the feasibility of HRV analyses in horses with colic and to analyze the correlation between parameters of the HRV analyses, serum cortisol concentrations, and the severity of the colic.

We hypothesized that horses with severe colic, in which a surgical intervention or euthanasia was required, would have higher stress level, higher serum cortisol concentrations, and lower variation in successive RR intervals translating in a reduction of the HRV variables of the time domain analysis and a lower HF value than horses with milder colic treated conservatively without surgical intervention and which survived. Moreover, we hypothesized that the HRV parameters of the time domain analyses and the HF values would increase over time in conservatively treated cases and in surgically treated cases that survived to discharge and would be equal in both groups on the day of discharge.

## 2. Materials and Methods

Study population—The data of 43 horses presented for acute abdominal pain (colic) in two equine referral hospitals between February and July 2012 were included in this study. Breed, use, age, and sex, as well as premedication (up to 2 h prior to arrival) and medication given after admission, were recorded and included in the analysis.

Data collection—All horses included in the study underwent a complete physical examination immediately after admission, focusing on the heart rate (HR), status of the circulatory volume and abnormalities in the gastrointestinal system. Furthermore, blood analyses, including packed cell volume (PCV), total protein (TP), and plasma lactate, rectal palpation, abdominal ultrasound, and passage of the nasogastric tube were performed. The clinical exam was completed by an abdominocentesis in 19 cases. The severity of colic pain was evaluated and graded as mild (low grade pain shown by pawing, flehmening, normal borborygmi, normal abdominal wall contour, mild distension at rectal examination), moderate (moderate grade of pain shown by lying down, diminished borborygmi, moderate distension of the abdominal wall contour, moderate distention at rectal examination), and severe (severe pain, absent borborygmi, severe distension of the abdominal wall contour and severe distention at rectal examination) [23]. An electrocardiogram (ECG) was recorded and the results of the blood samples were analyzed for the following time points: Upon arrival (T1), and, if horses were still alive or still in the hospital, between 9:00 a.m. and 11:00 a.m. on the day after the admission (T2), as well as on the day of discharge (T3). The initial treatment was based on findings of the clinical and laboratory examination and was adapted depending on case evolution by the clinician in charge, independently of the sampling scheme. The horses in the study were then retrospectively assigned to the following groups regarding the treatment applied or outcome: Group 1 = surgical, Group 2 = conservative, and Group 3 = euthanized (during hospitalization based on clinical worsening of the condition, poor prognosis or financial concerns). Group 1 and 2 were, therefore, discharged, and Group 3 represented the non-survivor group. Two horses were discharged on T2, and further measurement time points are consequently missing. Postsurgical medication was administered at the clinician’s discretion.

Blood samples analyzed in the present study were taken during routine puncture of the jugular vein. The PCV was measured by microcentrifugation and TP was measured with a handheld refractometer (HRM 18, 726000, Eickemeyer KG, Tuttlingen, Germany). The samples for plasma lactate measurements were collected in calcium-lithium-heparintubes (Arterial blood collection syringe, BD A-Line TM, 3 mL, Becton, Dickinson & Company, Plymouth, UK) and analyzed with a Cobas b 123 POC System (Roche Diagnostics Deutschland GmbH, Mannheim, Germany). The serum for the serum cortisol measurements was collected in plain tubes (Sarstedt AG & Co. KG, Nümbrecht, Germany). Subsequently, the samples were stored at −80 °C and later analyzed by high-performance liquid chromatography in a commercial laboratory (Laboklin GmbH & Co.KG, Bad Kissingen, Germany). According to the laboratory information, the analytic sensitivity was 2 ng/mL, the coefficient of variation for intra-assay variability was 9.92% and 2.39%.for inter-assay variance

All blood samples were taken for the routine work-up of colic patients in the hospitals. The results of the laboratory analyses (i.e., PCV, plasma lactate, TP) were used in the clinical work-up and in decision-making regarding the treatment of the horses. The results of these analyses were only statistically analyzed in the present study. The blood used for the cortisol analyses was leftovers from the samples taken for the routine clinical work-up. Therefore, according to the German law, sampling of horses affected by colic was not classified as animal experiments by the State Office of Health and Social Affairs Berlin (Landesamt für Gesundheit und Soziales)

The ECG was a surface ECG with adhesive electrodes placed on the horses. Therefore, it is a noninvasive procedure and according to the German law, was not classified as an animal experiments by the State Office of Health and Social Affairs Berlin (Landesamt für Gesundheit und Soziales). The ECG electrodes were placed on the horse in parallel to all the other examinations performed by a separate person not involved in the clinical work-up (M.-D.F.). Therefore, the recording of the ECG did not interfere or delay any of the decisions or procedures applied for the treatment of the horses enrolled. Lastly, owners’ verbal consent to involve their horses in the study was obtained during the admission process at both hospitals.

Analyses of the HRV—ECG recordings lasted 5 min (300 s) and were collected with a telemetric ECG device (Televet-100 EKG, Engel Engineering, Heusenstamm, Germany). The device was attached to the animal using four adhesive electrodes from which two were placed on the left chest wall at the elbow level and on the left chest wall at the withers level, representing a modified base-apex lead position [24]. A bandpass filter of 50 Hz was applied to avoid signal interference. The errors in RR waves recognition, arrythmias as supraventricular extrasystoles, associated with a compensatory shortened RR interval, atrioventricular blocks, and artifacts were corrected or removed manually from the ECG [24,25] using the dedicated software. The RR intervals were measured in milliseconds, exported from the reading software and imported into the HRV Analysis Software (HRV Analysis Software 2.0, Biosignal Analysis & Medical Imaging Group, Physics Department, University of Kuopio, Kuopio, Finland). This software allowed us to perform time and the frequency domain analyses. The time domain analysis is based on the analyses of the inter-beat interval, which is the time period between consecutive heartbeats (NN = Normal to Normal intervals). The parameters of the time domain analyses are the mean normal-to-normal (beat-to-beat) interval (meanNN), the standard deviation of NN intervals (SDNN), and percentage of consecutive RR intervals that differed more than 50 ms (pNN50). Moreover, HRV was analyzed in the frequency domain based on power spectral analysis using a fast Fourier transformation. The HRV parameters in the frequency domain were the high-frequency (HF) ranges, low-frequency (LF) ranges, and the low-/high-frequency ratio (LF/HF). The HF power shows mainly the vagal activity, whereas the LF power presented both vagal and sympathetic activity. The LF/HF ratio represented the sympathetic-vagal balance [21,22]. The frequency component thresholds for LF were set at 0.005–0.07 Hz, as previously described [20,21,22,26,27,28]. The upper limit for HF was set at 1.0 Hz due to the higher HF range in horses which is related to their respiratory rate, which might reached 60/min in some horses (personal communication with the Kubios HRV software engineer and suggested by Cottin et al. [29] and Rietmann et al. [19]. Therefore, the HF range was 0.07–1.0 Hz. The LF and HF were estimated in standard normalized units (n.u.) to allow the comparison between the measurements and patients.

Statistical analyses—Statistical analyses and graphical representations were performed in SPSS (IBM^®^-SPSS Inc., Chicago, IL, USA, Version 22.0) and Microsoft^®^ Excel (2010, Microsoft Corporation, Redmond, DC, USA). The normality of data distribution was verified with the Kolmogorov-Smirnov test. As the data were normally distributed, results were reported as mean and standard deviation (SD). Analyses of variance (ANOVA) tests were done first to assess the overall effect over time and between groups. Subsequently for multiple comparisons analyses using the Bonferroni correction were used. Furthermore, two-tailed Pearson correlation coefficients was calculated to assess the association between the HRV parameters and the HR, PCV, plasma lactate concentrations, and serum cortisol concentrations. The statistical significance level was established as *p* ≤ 0.05 as significant, *p* ≤ 0.01 as highly significant, and *p* ≤ 0.001 as most significant.

## 3. Results

### 3.1. Horses

The study population (*n* = 43) consisted of 19 mares, 23 geldings and 1 stallion. The breeds included 30 Warmbloods, 3 Haflinger horses, 3 Arabians, 2 Quarter horses, 1 Standardbred, 1 Frisian horse, and 1 Cold blooded horse. Ages of the horses ranged between 3 and 29 years (mean = 12 ± 6 years). Weight ranged between 250 and 680 kg (mean = 519 ± 88 kg) and height ranged between 144 and 178 cm (mean = 165 cm ± 9 cm). The demographic data for each group are summarized in Table 1. There was no statistically significant difference between the groups regarding age (*p* = 0.232), weight (*p* = 0.590) or height (*p* = 0.452).

Several horses had received treatments before referral and used drugs included Flunixin-Meglumine (9/43), Metamizole (16/43), Butylscopolamine (7/43), Xylazine (6/43) and Butorphanol (4/43). One of the horses (1/43) was treated with Flunixin-Meglumine (1.1 mg/kg every 8 h), Amoxicillin (10 mg/kg every 12 h and Gentamicin (6.6 mg/kg every 24 h for reasons unrelated to colic.

Routine treatment protocol in the post-operative period consisted of administration of Flunixin-Meglumine 1.1 mg/kg every 8 h, Amoxicillin 10 mg/kg every 12 h, Gentamicin 6.6 mg/kg every 24 h, Ringer Lactate (varying amount to cover maintenance and eventual deficits) and Lidocaine (3 mg/kg/h, bolus followed by CRI at 0.05 mg/kg/min, for 48 h or until cessation of reflux), and Heparin (120 IE/kg every 12 h). The exact regime varied depending on the intraoperative diagnosis and intervention (i.e., resection performed or not), on the clinical signs present, and the clinician’s assessment. Five horses additionally received Sulfadiazin-Trimethoprim (30 mg/kg every 12 h) and Meloxicam (0.6 mg/kg every 24 h) due to wound healing complications or Sulfadiazin-Trimethoprim (30 mg/kg every 12 h) and Heparin (80 IE/kg every 24 h) due to peri-/thrombophlebitis.

### 3.2. HRV Analyses

The results of the HRV parameters are presented in Table 2 and Table 3. The meanNN increased over time in group 1 and 2 in the time domain analyses, however reached significant levels only in group 1 at T3. These changes in meanNN translated into a significantly decreasing HR in both groups. In group 3, however, there was no significant change over time. Furthermore, the meanNN and HR differed significantly in group 3 compared to the other two groups at T2. At T1, group 3 showed significantly different values than group 2, whereas the difference between group 1 and 3 was less evident at T1. The most visible changes over time in the frequency domain analysis, occurred in group 1, whereas the largest difference between groups was seen at T1, where group 2 showed a significant difference to group 1 and 3. Therefore, there was a significant evolution over time in the HRV parameters in horses that were discharged (Group 1 and 2) but not in the group 3 representing the non-survivors.

### 3.3. Laboratory Variables

Serum cortisol concentrations also decreased over time in all groups, but the difference did not reach statistical significance for group 3. They were significantly lower in group 2 in comparison to group 1 and 3 at T1. However, the difference between group 2 and 3 was not significant at T2. Plasma lactate values also decreased over time in all groups, and the difference between group 3 and group 2 or 1 was significant at T1, as well as at T2 (Table 4).

### 3.4. Correlation

The two-tailed Pearson correlation revealed significant correlations between some of the HRV parameters and PCV, plasma lactate, and serum cortisol values (Table 5). There was no significant correlation between the TP and HRV parameters.

## 4. Discussion

The main conclusion of this study is that the HRV measurements on the short-time ECG (5 min sequences) can be accomplished in horses with colic and that the changes in the HRV parameters and serum cortisol concentrations showed a decreased sympathetic simulation with treatment. This was particularly visible in the increased meanNN and the HF power over time in the groups surviving to discharge. Furthermore, it was visible that the meanNN was the only variable of the time domain analysis that changed significantly over time and between groups, whereas the other variables of the time domain analyses did not add any further information. The SDNN, especially, did not appear to be a useful variable in our research question. Similarly, the European Task Force concluded that the SDNN was a very imprecise variable for the characterization of HRV, especially for the short ECG procedure, since the chronology of NN intervals is not included in the analysis, and a distinction between the random changes and regular processes of the variability cannot be made [12]. However, the meanNN paralleled the results of the HR and did not add further information about the HR in the clinical assessment of the patient. Results in the frequency domain analyses were similar to the time domain analyses at time point T1 and T2 but not at T3. The advantage of the frequency domain analysis is to generate information which cannot be obtained by the time domain parameters. The frequency domain analyses in human medicine highlight the changes in the sympathovagal balance and allows one to better distinguish between the sympathetic and parasympathetic activity [12]. However, in our study, there was no obvious advantage of the frequency domain analysis over the time domain analysis, as the evolution over time was less consistent, even if group 2 at T1 had significantly different values than the other two groups for HF, LF, and for LF/HF.

The difficulties encountered in the interpretation of the results from the study presented were mainly due to the many factors that might influence the HRV and that could not be standardized further in our study. Firstly, all horses included in the study were premedicated with different drugs and postsurgical treatments were not standardized. The drugs applied could, therefore, have influences on the HRV. It can generally be assumed that the anti-inflammatory and sympatholytic agents increase the HRV [19,22], whereas the vagal nerve-inhibiting agents decrease the HRV [22]. Many horses were treated with analgesic drugs (Metamizole, Flunixin-meglumine, Butorphanol, or Xylazine) and spasmolytic drugs (Butylscopolamine) by the referring veterinarians prior to admission to the hospital. The drug elimination half-life (also the effect-time) of Metamizole [30], Flunixin [31,32], and Butorphanol [33] last several hours; therefore, upon arrival to the hospital, the HRV was probably influenced by the first two drugs, whereas the opioids had less effect on the HRV according to a study in humans [34]. Xylazine is a short-active a2-agonist [35,36] with secondary vagolytic effects. Due to the short-lasting effect of xylazine, this drug had probably few impacts on the HRV during the first examination. Butylscopolamine is also a vagolytic drug and was used in nearly all horses by the referring veterinarians. Butylscopolamine reduced the HF power in one equine study [37], whereas several other studies [38,39,40] in humans have shown that at a low dosages, muscarine receptor blockers can paradoxically increase the vagal activity. However, Butylscopolamine is a short-acting [41] drug, and it probably had no effect on the HRV at the time of admission.

Furthermore, the drugs used during the induction and maintenance of anesthesia on horses in group 1, specifically Medetomidine, Ketamine, Diazepam, and Isoflurane, might also have had a negative influence on the vagal activity [42]. However, the duration of the drug effects is also short-lasting [33,43,44,45]; thus, these medications probably had no effect on the HRV at T2. At this time point, the horses that had been to surgery were treated with Flunixin-meglumine (1.1 mg/kg, IV, every 8 h), which also had a positive influence on the HRV and a long-lasting effect. Therefore, Flunixin-meglumine might also have influenced the HRV parameters. The effect of Lidocaine, that was given in horses in the group 1 and 3, on the HRV parameter is not well studied. It is a class 1b antiarrhythmic drug and would probably have the same inhibiting effects on the vagal nerve as class 1c antiarrhythmic drugs (e.g., Fecainid or Propafenone). However, the HRV parameters could also be influenced by the anti-inflammatory attributes of Lidocain [46]. Similarly, the influence of the antibiotics on the HRV parameters remains unclear. Antibiotics (Penicillin 30.000 IU/kg, IV, every 6 h; Gentamicin, 6.6 mg/kg, IV, every 24 h) were used to treat the surgical patients in groups 1 and 3 (at T2) and the patients in group 1 with wound healing problems or thrombophlebitis (at T3). Horses with thrombophlebitis were also treated with heparin, which probably has a positive influence on the HRV due to its thrombolytic effect [47,48]. Furthermore, these two patients with thrombophlebitis received Meloxicam, which increases the HRV because of its analgesic effect [19,22]. Even if the drugs mentioned might have had some influence on the HRV, it was outside the scope of this study to assess their effect.

Another important limitation of the study is the inhomogeneous age, sex, and breed of the horses. However, according to Nagel et al., there was no difference in the HRV between warmbloods and ponies [49]. The literature provides further controversial results on the influence of age or sex on the HRV in animals. A study in horses has documented an influence of sex on the HRV parameters [50], whereas some studies in dogs have not found any relationship between sex and HRV parameters [51,52]. The influence of age on HRV parameters in humans was linked to pulse rigidity that increased with age [53]. In horses, conflicting results have been reported for the effect of age. In an older study, age was found to have had a small but significant influence on the LF specters of the HRV parameters [50]. Lower vagal activity measured in the frequency domain analyses has, for example, been shown in foals and yearlings in comparison to older horses [54,55,56]. By contrast, a newer study comparing horses between 4 and 6 years of age and horses >22 years of ages found a higher HR and lower meanNN in the older horses [57]. However, another study did not find any significant influence of age on the HRV in horses [19]. Unfortunately, the training effect in the group of younger horses in many of these studies might have influenced the results [54,55,56,57]. In the current study, all horses were adult (mean age 12 ± 6 years). Furthermore, even if horses in group 3 seemed to be older, there was no statistically significant difference between the groups, and, therefore, age would be expected to have had relatively little influence on our population. Lastly, the temperament of the horses will certainly have had an influence on the HRV [58,59,60,61] but could not be assessed in the current study.

Concerning environmental factors, it is known that the HRV has a diurnal rhythm [56,60,62], and, therefore, the recordings at T2 and T3 were done in the morning. The examination environment, however, was not ideal. Upon arrival (T1), the horses were examined in the examination room, directly after transportation, surrounded by many people, whereas the examinations at T2 and T3 were performed in quiet rooms with only two people being involved. The European Task Force recommended a quiet surrounding during the admission examination to avoid such influences [12], and this should certainly be applied in future studies. The feeding regime also changed between day 2 and day 3, when horses were starved on day 2 and fed on day 3; however, this should not influence the HRV [63,64].

However, the results of the HRV analyses paralleled the results of the cortisol concentration. Furthermore, there was a positive correlation between cortisol concentrations and LF/HF. Higher cortisol level was, therefore, associated with lower HF values, higher LF or both, indicating a reduced parasympathetic activation, as it is expected in painful conditions. Similarly, PCV and plasma lactate concentrations also correlated negatively with parameters of the HRV. Whereas PCV showed no difference in the three groups or between the three time points, the plasma lactate concentrations allowed to distinguish group 3 from the other two groups and T1 and T2. Both lactate and PCV are parameters indicating the status of the circulatory volume and, therefore, are related to the severity of fluid loss in the condition. Fluid loss is a powerful drive for stimulation of sympathetic system and is, therefore, negatively related to the variation if the RR intervals or the HF power.

## 5. Conclusions

As there are so many factors that influence the HRV, it is essential to standardize the registration environment, handling, and drugs applied as much as possible. However, even with this many influencing factors present, we still could demonstrate that treatment induced a change in the HRV and cortisol response in horses managed conservatively or surgically but not in horses that required euthanasia. Furthermore, cortisol concentrations correlated positively with LF/HF. Therefore, we concluded that the HRV can provide further important information on the stress level in horses with colic and might be helpful in assessing possible outcome. However, further studies are required to assess the validity of the HRV analyses in horses with colic. Another interesting finding was that plasma lactate was the only variable that constantly differed in group 3 from group 1 and 2, underscoring the importance of evaluating plasma lactate in the assessment of the severity of diseases in horses with colic [9,10].

## Figures and Tables

**Table 1:** Demographic data of the study population, including age, sex, breed, weight and height, and athletic use of the horses in each treatment group.

Group	Age (y) Mean ± SD	Sex m/g/s *	Breed wb/others **/pony/hh	Weight (Kg) Mean ± SD	Height (cm) Mean ± SD	Athletic Use p/b/l/s ***
1 (*n* = 11)	12 ± 6	4/6/1	9/1/0/0	506 ± 69	165 ± 9	1/2/3/5
2 (*n* = 24)	12 ± 6	9/15/0	15/7/1/1	532 ± 84	165 ± 9	1/0/14/9
3 (*n* = 8)	16 ± 7	6/2/0	5/2/1/0	500 ± 124	159 ± 18	1/1/6/0
all (*n* = 43)	12 ± 6	19/23/1	30/10/2/1	519 ± 88	164 ± 11	3/3/23/14

* m, mare/g, gelding/s, stallion; ** others: 3 Arabian, 3 Haflinger, 2 Quarter horses, 1 Frisian horse, 1 standardbred. *** b, breeding/l, leisure/p, paddock/s, sport (dressage, show jumping, western); hh, heavy horse; Kg, Kilogram; 2x; SD, standard deviation; wb, warmblood; y, years.

**Table 2:** Heart rate variability (HRV) parameters in the time domain analysis at the three measurement time points (T1, T2, and T3) and in the three treatment groups.

		T1	T2	T3		
	**Group**	**Mean ± SD** **(Number)**	**Mean ± SD** **(Number)**	**Mean ± SD** **(Number)**	***p-Value for the Overall ANOVA***	***p-Values between Time Points for the Bonferroni’s Multiple Comparison***
**HR** (bpm)	**1**	60 ± 11 (*n* = 11)	45 ± 7 (*n* = 11)	38 ±- 5 (*n* = 11)	*<0.001*	*T1-T2: 0.001* *T1-T3: <0.001*
	**2**	45 ± 9 (*n* = 24)	37 ± 6 (*n* = 23)	38 ± 3 (*n* = 10)	*0.003*	*T1-T2: 0.004* *T1-T3: 0.042*
	**3**	67 ± 28 (*n* = 8)	60 ± 20 (*n* = 4)	-	*0.680*	-
	*p-Value for the Overall ANOVA*	*0.001*	*<0.001*	*0.923*		
	*p-Values between Groups for the Bonferroni’s Multiple Comparison*	*1–2: 0.024* *2–3: 0.002*	*1–2: 0.036* *1–3: 0.011* *2–3: <0.001*	-		
**MeanNN** (ms)	**1**	1216.94 ± 352.37 (*n* =10)	1370.61 ± 126.71 (*n* = 11)	1577.72 ± 186.61 (*n* = 11)	*0.006*	*T1-T3: 0.005*
	**2**	1489.82 ± 305.77 (*n* = 24)	1684.53 ± 276.42 (*n* = 23)	1605.93 ± 220.35 (*n* = 10)	*0.067*	-
	**3**	940.76 ± 328.11 (*n* = 8)	969.73 ± 278.32 (*n* = 4)	-	*0.883*	-
	*p-Value for the Overall ANOVA*	*<0.001*	*<0.001*	*0.753*	-	
	*p-Values between Groups for the Bonferroni’s Multiple Comparison*	*2–3: <0.001*	*1–2: 0.004* *1–3: 0.024* *2–3: <0.001*	-		
**SDNN** (ms)	**1**	120.38 ± 120.07 (*n* = 10)	61.24 ± 24.67 (*n* = 11)	62.29 ± 32.00 (*n* = 11)	*0.114*	*-*
	**2**	70.84 ± 33.31 (*n* = 24)	80.58 ± 45.06 (*n* = 23)	87.25 ± 34.20 (*n* = 10)	*0.479*	*-*
	**3**	56.14 ± 49.06 (*n* = 8)	28.03 ± 23.55 (*n* = 4)	-	*0.311*	*-*
	*p-Value for the Overall ANOVA*	*0.088*	*0.050*	*0.100*		
	*p-Values between Groups for the Bonferroni’s Multiple Comparison*	-	-	-		
**pNN50** (%)	**1**	30.88 ± 34.16 (*n* = 10)	30.86 ± 16.04 (*n* = 11)	22.39 ± 15.82 (*n* = 11)	*0.622*	-
	**2**	29.75 ± 18.86 (*n* = 24)	33.15 ± 17.73 (*n* = 23)	37.08 ± 14.30 (*n* = 10)	*0.532*	-
	**3**	14.99 ± 23.31 (*n* = 8)	4.95 ± 9.64 (*n* = 4)	-	*0.436*	-
	*p-Value for the Overall ANOVA*	*0.287*	*0.013*	*0.050*		
	*p-Values between Groups for the Bonferroni’s Multiple Comparison*	-	*1–3: 0.035* *2–3: 0.011*	*-*		

Bpm, beats per minute; HR, heart rate; meanNN; mean normal to normal R intervals, SD, standard deviation; SDNN, the standard deviation of NN intervals; pNN50, percentage of consecutive R-R intervals that differed more than 50 ms. *p* values are indicated in italic

**Table 3:** HRV parameters in the frequency domain analyses at the three measurement time points (T1, T2, and T3) and in the three treatment groups.

			T1	T2	T3		
	**Group**		**Mean ± SD** **(Number)**	**Mean ± SD** **(Number)**	**Mean ± SD** **(Number)**	***p-Value for the Overall ANOVA***	***p-Values between Time Points for the Bonferroni’s Multiple Comparison***
**HF** (n.u.)	**1**		18.47 ± 41.9 (*n* = 10)	49.22 ± 19.98 (*n* = 11)	29.44 ± 12.44 (*n* = 11)	*0.002*	*T1-T2: 0.002* *T2-T3: 0.049*
	**2**		37.43 ± 15.61 (*n* = 24)	43.01 ± 14.55 (*n* = 23)	43.07 ± 12.45 (*n* = 10)	*0.371*	*-*
	**3**		19.99 ± 14.74 (*n* = 8)	36.20 ± 23.08 (*n* = 4)	-	*0.165*	*-*
	*p-Value for the Overall ANOVA*		*0.005*	*0.391*	*0.021*		
	*p-Values between Groups for the Bonferroni’s Multiple Comparison*		*1–2: 0.015* *2–3: 0.047*	-	*1–2: 0.0231*		
**LF** (n.u.)	**1**		81.53 ± 21.22 (*n* = 10)	50.78 ± 19.98 (*n* = 11)	70.56 ± 12.44 (*n* = 11)	*0.002*	*T1-T2: 0.002* *T2-T3: 0.049*
	**2**		62.57 ± 15.62 (*n* = 24)	56.98 ± 14.56 (*n* = 23)	48.03 ± 12.45 (*n* = 10)	*0.371*	*-*
	**3**		79.9 ± 15.0 (*n* = 8)	63.80 ± 23.08 (*n* = 4)	-	*0.171*	*-*
	*p-Value for the Overall ANOVA*		*0.006*	*0.391*	*0.021*		
	*p-Values between Groups for the Bonferroni’s Multiple Comparison*		*1–2: 0.015* *2–3: 0.050*	-	*1–2: 0.021*		
**LF/HF**	**1**		7.17 ± 3.15 (*n* = 10)	1.48 ± 1.31 (*n* = 11)	3.03 ± 1.83 (*n* = 11)	*<0.001*	*T1-T2: <0.001* *T1-T3: <0.001*
	**2**		2.34 ± 2.02 (*n* = 24)	1.68 ± 1.24 (*n* = 23)	1.68 ± 1.48 (*n* = 10)	*0.338*	-
	**3**		6.98 ± 5.93 (*n* = 8)	3.68 ± 4.60 (*n* = 4)		*0.355*	-
	*p-Value for the Overall ANOVA*		*< 0.001*	*0.108*	*0.81*		
	*p-Values between Groups for the Bonferroni’s Multiple Comparison*		*1–2: 0.001* *2–3: 0.004*	-	-		

HF, high-frequency ranges in the frequency domain analysis; LF, low-frequency ranges in the frequency domain analysis; LF/HF, low-/high-frequency ratio; n.u., normalized units (n.u.) SD, standard deviation. *p* values are indicated in italic.

**Table 4:** Laboratory variables at the three measurements time points (T1, T2, and T3) and in the three treatment groups.

		T1	T2	T3		
	**Group**	**Mean ± SD** **(Number)**	**Mean ± SD** **(Number)**	**Mean ± SD** **(Number)**	***p-Value for the Overall ANOVA***	***p-Values between Time Points for the Bonferroni’s Multiple Comparison***
**Serum Cortisol** (mmol/l)	**1**	119.8 ± 41.9 (*n* = 11)	97.1 ± 46.3 (*n* = 11)	47.1 ± 20.5 (*n* = 11)	*<0.001*	*T1-T3: <0.001* *T2-T3: 0.013*
	**2**	82.2 ± 40.0 (*n* = 24)	57.3 ± 16.2 (*n* = 23)	51.1 ± 16.8 (*n* = 10)	*0.004*	*T1-T2: 0.014* *T1-T3: 0.018*
	**3**	127.1 ± 67.9 (*n* = 8)	71.9 ± 41.4 (*n* = 4)	-	*0.143*	-.
	*p-Value for the Overall ANOVA*	*0.024*	*0.004*	0.628		
	*p-Values between Groups for the Bonferroni’s Multiple Comparison*	*1–2: 0.032* *2–3: 0.023*	*1–2: 0.001*	-		
**PCV** (%)	**1**	36 ± 7 (*n* = 11)	32 ± 7 (*n* = 11)	30 ± 4 (*n* = 11)	0.075.	-
	**2**	34 ± 6 (*n* = 24)	32 ± 3 (*n* = 23)	32 ± 3 (*n* = 10)	0.393	-
	**3**	43 ± 18 (*n* = 8)	36 ± 11 (*n* = 4)		0.445	-
	*p-Value for the Overall ANOVA*	*0.047.*	*0.459*	*0.235*		
	*p-Values between Groups for the Bonferroni’s Multiple Comparison*	*2–3: 0.042*	-.	-		
**TP** (g/dl)	**1**	6.6 ± 1.1 (*n* = 11)	5.5 ± 0.6 (*n* = 11)	6.4 ± 0.7 (*n* = 10)	*0.017*	*T1-T2: 0.022*
	**2**	6.6 ± 0.7 (*n* = 24)	6.3 ±0.7 (*n* = 22)	6.4 ± 0.7 (*n* = 10)	*0.456*	-.
	**3**	5.6 ± 1.9 (*n* = 7)	4.3 ± 1.1 (*n* = 4)		*0.238.*	-.
	*p-Value for the Overall ANOVA*	0.082	*<0.001*	*1.00*		
	*p-Values between Groups for the Bonferroni’s Multiple Comparison*	-	*1–2: 0.019* *1–3: 0.013* *2–3: <0.001*	-		
**Plasma Lactate** (mmol/l)	**1**	2.1 ± 1.3 (*n* = 11)	1.1 ± 0.3 (*n* = 11)	1.0 ± 0 (*n* = 10)	*0.003*	*T1-T2: 0.014* *T1-T3: 0.006*
	**2**	1.3 ± 0.4 (*n* = 24)	1.0 ± 0.1 (*n* = 23)	1.1 ± 0.2 (*n* = 9)	*0.019*	*T1-T2: 0.022*
	**3**	5.9 ± 5.3 (*n* = 7)	2.1 ± 0.9 (*n* = 4)		*0.205*	-.
	*p-Value for the Overall ANOVA*	*<0.001*	*<0.001*	*0.281*		
	*p-Values between Groups for the Bonferroni’s Multiple Comparison*	*1–3: 0.003* *2–3: <0.001*	*1–3: 0.001* *2–3: 0.001*	-.		

SD, standard deviation. *p* values are indicated in italic.

**Table 5:** Correlation coefficients (*r*) with significances (*p*, *p*-value) between packed cell volume (PCV), plasma lactate concentration, serum cortisol concentration, and the HRV parameters mean normal to normal R intervals (meanNN), percentage of consecutive RR intervals that differed more than 50 ms (pNN50), high-frequency (HF) ranges in the frequency domain analyses, low-frequency (LF) ranges in the frequency domain analyses, and the low-/high-frequency ratio (LF/HF).

Parameter		PCV	Plasma Lactate	MeanNN	HF	LF	LF/HF
**PCV**	*r*		0.202	−0.406	−0.300	0.300	0.459
	*p*		0.200	0.008	0.054	0.054	0.002
**Plasma Lactate**	*r*			−0.537	−0.339	0.339	0.476
	*p*			<0.001	0.030	0.030	0.002
**Serum Cortisol**	*r*	0.542	0.361				0.396
	*p*	<0.001	0.019				0.010
